# Report of the Meeting of the Pennsylvania State Dental Society

**Published:** 1883-10

**Authors:** W. H. Trueman


### REPORT OF THE MEETING OF THE PENNSYLVANIA STATE DENTAL SOCIETY, HELD AT CRESSON, PA., JULY 31, AND AUGUST 1 AND 2, 1883.

BY W. H. TRUEMAN, D. D. S.

### THE FIRST DAY, JULY 31ST. MORNING SESSION.

The meeting was called to order by the President, Dr. J. C. Green, of West Chester, at 11 o'clock.

The Rev. Mr. Russell, of Altoona, delivered an address of welcome, which was responded to by Dr. Gerhart, of Lewisburg. The rest of the morning session was devoted to routine business.

### AFTERNOON SESSION.

The President delivered his address, taking as his theme the manner in which advances in science are made. He endeavored to show how much valuable time and earnest effort is constantly wasted by being improperly directed. Would be investigators neglect to ascertain how far the subject they are interested in has been studied, or attempt to unravel the deeper mysteries of a science before they have mastered its first principles, or attempt a little here and a little there without any method or fixed object in view. In each case the result is a waste of time and effort. We should consider not only what we already know, but also what we desire to know, so that each effort shall be directed to a definite object.

It has been well said, "He who sees most and comprehends what he sees, makes the best use of his time." It is not sufficient to simply *acquire* knowledge, or by constant practice acquire skill, but what we need most is the power to make that knowledge and skill practical. A man may be ready with his knowledge, be ready to describe and explain the various diseases and their best mode of treatment, and yet not recognize symptoms when he sees them, and be entirely at a loss in selecting a remedy. He may be a good mechanic, and yet failing to exercise judgment, be a poor workman. We should study to see not only that which lies upon the surface, but also that which is hidden deep within, and constantly bear in mind that all knowledge to be useful must be practical.

The doctor closed his address with an earnest plea for scientific, persistent, methodic work, in unraveling the mystery surrounding the cause of dental caries, as a ground-work to a better understanding of its prevention and relief.

Dr. James Truman read a paper upon "Iodoform in Dentistry." He had noticed in the foreign journals frequent reference to the use of this substance in dentistry, especially in Germany. Seeing it highly recommended by men whom he personally knew to be competent and reliable, he was induced to give it a careful and guarded trial. It seemed to be but little known in this country, and believing that it possessed merit he had taken this opportunity of calling attention to it. So far the results had been very satisfactory, but he had not been using it long enough to feel able to estimate its real value.

Iodoform is an orange colored, fine crystaline powder, with a strong persistent saffron odor. To many the odor was quite unpleasant, so much so that it has been cited as an objection to its use, and is said in some cases to cause severe headache. He had used it carefully and had had very little complaint of it on that ground. It is desirable to keep it off the hands, the odor being more difficult to remove than that of creasote.

It has three properties that render it desirable to us as dentists. It is antiseptic; it allays pain; its power to give prompt and permanent relief is remarkable. It is a disinfectant, and seems to possess the power of uniting with the gases given off during decomposition, and rendering them inert. It possesses these properties to a far greater degree than anything he was acquainted with. A knowledge of these properties will suggest the cases where it can be used to advantage. It seems to combine the action of chloroform and iodine, but is far more permanent in its effect than either. For convenience of application he mixed it with a ten per cent solution of carbolic acid, using about the one-twentieth of a grain of iodoform. It is very important to thoroughly seal it up, so that it cannot possibly mingle with the saliva ; he thought it quite as important to do this in using iodoform as in using arsenic, for it must not be forgotten that this substance is a poison, minute doses sometimes producing headache, nausea, and other unpleasant symptoms.

In illustrating its practical use, he stated that in a case of severe toothache with an exposed or nearly exposed pulp, he would proceed much in the same way as though he intended to make an arsenical application, only using more care in excavating. Then he would take about the one-twentieth of a grain of iodoform, moisten it with creasote solution, and carry it to the previously well-dried cavity on a small pledget of cotton—just as he would the arsenic paste—and carefully seal with cotton and sandarach varnish, using the same care to avoid pressure and to perfectly seal the cavity that he would in using arsenic. Usually the pain ceased in a few moments and did not return. He would leave that application undisturbed for a week or ten days, if the tooth remained comfortable. On the patient's return, after adjusting the rubber dam and preparing the cavity, he would place an application of iodoform over the point of exposure, and leaving it there proceed to cap the pulp by any of the usual methods. What the final condition of that pulp would be he could not tell. He could only say that with but few exceptions they have remained comfortable, and nearly all the cases have been those where in his judgment any attempt to save the pulp by the usual methods would have failed.

He had also found it useful in cases where the pulp was dead and putrescent. In these cases, after cleaning the cavity and opening well into the roots, he would pass a little cotton saturated with the iodoform and creasote down the canal, and seal it up. It seldom required a second application.

In conclusion, he enjoined extreme care in its use, quite as much as we now recognize is required in using arsenic. He believed that used with judgment it would prove a valuable acquisition.

DISCUSSION.

Dr. C. N. Pierce had found it of value in treating putrescent pulps, and also in cases of pulp irritation. Could a tooth with an exposed pulp be filled over it with safety to the pulp? This is an important question that his experience had failed to answer.

Dr. E. T. Darby thought that from some cause they were more successful in treating these cases in Germany than here. He recently read an article in a German journal in which the writer claimed to have had only six failures in treating one hundred cases of nerve exposure. That was a far larger percentage of success than is usually met with here. He had treated a few cases of irritated pulps by sprinkling a little iodoform powder over the point of exposure, covering it with paper, and then sealing it up with gum-damar and wax. The pain almost immediately ceased, and ten days after he renewed the application, this time using a solution of it in glycerine and carbolic acid, and filled temporarily. They had remained perfectly easy, but of the final result he could not speak.

Dr. James Truman said that in cases of recession of the gum, and consequent irritation of the periosteum, he had found almost immediate relief on application of iodoform in solution. Several gentlemen stated they had used it with good results, but all were in doubt as to the ultimate effect upon the pulp, whether it really restored it to a healthy condition or whether the immunity from pain was owing to its powerful antiseptic and disinfectant properties, acting as the chloride of zinc in the oxy-chloride cement sometimes does when the pulp becomes mummified. All had found it a quick and lasting pain obtunder.

Dr. Guilford related a case where it had been applied after a severe surgical operation for the relief of hemorrhoids, by sprinkling the powder over the wounded surface. It eased the pain quickly, and the patient rode to his home several miles distant over a rough country road, with comfort. Several cases were related where the application had been followed by severe headache and nausea, supposed to have been caused by not thoroughly sealing the cavity.

EVENING SESSION.

Dr. William H. Trueman read a paper, entitled "The Screw in Regulating." (See page 521). He exhibited an upper cast with both canines presenting immediately behind the laterals. In this case the teeth were so short that in order to get the bands to hold firmly he fitted the bands to the teeth in the mouth, running them well under the gums. Then he took an impression with plaster while the bands were on the teeth; this is not often necessary. The screw was placed between the two teeth, working upon both at once. In two weeks they were in place without having been at all sore, or causing the patient any great inconvenience.

In another case, the lower incisors antagonized outside of the upper teeth. A screw with a loop at each end had brought them into position.

Another, where the lateral was very much inside the arch, had been corrected in a few days.

DISCUSSION.

Dr. Magill favored a plate wherever it could be used. It distributed the strain better, he thought, and gave a better chance to readily change the direction of force. In connection with plates he used either wedges or springs. He had good results in moving teeth out by taking an impression and, after making the cast, scraping all the teeth a little at the neck, and the tooth to be moved considerable. On this cast he made a simple vulcanite plate, leaving it a little thicker opposite the tooth to be moved. When made the plate fitted in tight, and pressed very hard against the misplaced tooth. After a few days, when the tooth had moved out a little, he warmed the plate opposite it and with the pliers pressed it out so that it would again press hard against it, repeating this operation as often as needful. The smooth plate proved but little obstruction in the mouth, and formed an effective arrangement.

Dr. Guilford appreciated the value of the screw, and had used it in much the same way that Dr. Trueman had described, except that he preferred to make the bands wide and thin, and instead of soldering tongues to them he made sockets into which the nuts of the screw fitted. He then cemented the bands to the teeth with either oxy-chloride or oxy-phosphate cement. He had found that bands put on in this way and kept dry for five or ten minutes, held very firmly. When the cement was hard he slipped the screw-jack in the sockets and screwed it up tight. It was less labor, he thought, than soldering on tongues and soldering the screw-jacks to them. He had arranged them entirely by the mouth, without making a cast. He also thought, not being rigidly fastened to the bands a decided advantage. It allowed the band on the tooth to be moved to be placed nearer the crown, and effectually prevented any injury to the gums. In the plan suggested by Dr. Trueman, and shown on the casts he exhibited, the band is placed at the neck of the tooth, where the force used acts with the least advantage. That he had been able to accomplish the results shown demonstrated the effectiveness of the screw. When he read the article of Dr. Farrar, referred to by Dr. Trueman, he had been much impressed with it, but since then he had been led to doubt the value of an intermittent force, and was inclined think the freedom from pain and irritation was owing to the care taken to keep the appliance from pressing into and irritating the soft tissues. In Dr. Farrar's appliances, and also in those shown by Dr. Trueman, there would be very little risk of any undue pressure upon the soft parts, and therefore but little irritation. Since the idea had been suggested to him by Dr. Magill, several years ago, he had abandoned ligatures and in their place used thin platina or gold rings cemented to the teeth, soldering to them hooks or rings to which he secured springs cut from rubber tubing. These he was able to fix just where they were needed, and by securing them near the cutting edge, the springs rested on the teeth alone. Since he had adopted this plan he had scarcely any trouble from excessive soreness of the teeth or gums. He had recently moved back a bicuspid in a case where he had extracted the first molar. In that case he made a ring to fit the molar and soldered a hook pointing back on each side, and fitted a similar ring to the bicuspid. When they were both in place, he stretched a ring cut from regulating tube, on each side, simply slipping it over the hooks. It worked quite rapidly and with very little pain. These rings may be kept in place as long as needed; they very seldom give way, and will be found very useful for many purposes, and they are quickly made. He took a strip of gold or platina, preferring the latter, and fitted it to the tooth in the mouth with a burnisher. Then marking with a point where the ends lap, removed and soldered it. For hooks he takes the headed pins from a vulcanite tooth, and holding them in position with a little plaster soldered them. Then cuts off the extra length and bends them into a hook. He had one objection to the arrangement shown by Dr. Trueman; he noticed that the fixed end of the screw was attached to only one tooth. No matter how firmly fixed the tooth might be, the strongest molar he had ever seen, he would never trust it alone, unless it was a case where very little force was needed. He always distributed the strain at that point between two or three teeth. He would never think of doing as was done in the case shown, attempt to draw in a canine with the other end of the screw fixed to a single molar. He thought there was great risk of injury, and preferred to be on the safe side.

Dr. Truman in reply said,—In the case referred to, while the screw was only fixed to one tooth, the direction of strain was such that the tooth was supported by all the teeth in front of it on that side. In that case, as in the others exhibited, any addition to the band at that point would have been useless. In the case in question all six of the lower front teeth were so far out that they antagonized outside of the upper teeth. The arrangement shown had been used to draw in the lateral and central of that side; a similar arrangement had been used on the other side, and the teeth had been successfully brought into position. The molar had resisted the strain of bringing in both teeth at once, and had sustained no injury so far as he could see. The arrangement shown had been altered to act upon the canine, but before it could be applied the patient was taken sick, and when she next presented, some six months after, the canines had so far changed their position that it was deemed best to let them alone. The teeth had proved very difficult to regulate, owing, no doubt, to the patient's age, about twenty-seven. In some cases it is best to include several teeth, but in others it would only complicate matters. In regard to cementing the bands in place, he doubted whether it would answer. It might if they were made with sockets, as Dr. Guilford suggested. That was a good idea, if practical. He had tried it with the bands soldered to the screws, and it did not hold. He had tried to hold the bands in place with ligatures of gilling twine, wire, etc., but they would not hold against the force needed to turn the screw. He had used bands such as described in many cases, and always with success. The space required by the bands described by Dr. Guilford were an objection to their use in very crowded mouths, and also, the teeth did not slide over them as readily as they would over each other. The narrow bands at the necks of the teeth took up no room, and if carefully arranged could be made to act as wedges, so as to facilitate the teeth passing each other. There are many cases where bands cemented on the teeth were very useful. Dr. Guilford had met the suggestion several years ago, and he had frequently used it to advantage.

(To be concluded in November No.)

